# Social Determinants of Health, Race, and Diabetes Population Health Improvement: Black/African Americans as a Population Exemplar

**DOI:** 10.1007/s11892-022-01454-3

**Published:** 2022-03-03

**Authors:** Felicia Hill-Briggs, Patti L. Ephraim, Elizabeth A. Vrany, Karina W. Davidson, Renee Pekmezaris, Debbie Salas-Lopez, Catherine M. Alfano, Tiffany L. Gary-Webb

**Affiliations:** 1 Institute of Health System Science, Feinstein Institutes for Medical Research at Northwell Health, 130 E 59th St, Ste 14C, New York, NY 10022 USA; 2 Department of Community and Population Health at Northwell Health, Manhasset, NY USA; 3 Institute of Cancer Research, Feinstein Institutes for Medical Research at Northwell Health, NY Manhasset, USA; 4 Department of Epidemiology, University of Pittsburgh Graduate School of Public Health, Pittsburgh, PA USA

**Keywords:** Social determinants of health, Health care disparities, Health care inequalities, Racial minorities, Population health, Diabetes

## Abstract

**Purpose of Review:**

To summarize evidence of impact of social determinants of health (SDOH) on diabetes risk, morbidity, and mortality and to illustrate this impact in a population context.

**Recent Findings:**

Key findings from the American Diabetes Association’s scientific review of five SDOH domains (socioeconomic status, neighborhood and physical environment, food environment, health care, social context) are highlighted. Population-based data on Black/African American adults illustrate persisting diabetes disparities and inequities in the SDOH conditions in which this population is born, grows, lives, and ages, with historical contributors. SDOH recommendations from US national committees largely address a health sector response, including health professional education, SDOH measurement, and patient referral to services for social needs. Fewer recommendations address solutions for systemic racism and socioeconomic discrimination as root causes.

**Summary:**

SDOH are systemic, population-based, cyclical, and intergenerational, requiring extension beyond health care solutions to multi-sector and multi-policy approaches to achieve future population health improvement.

## Introduction

Diabetes is a substantial contributor to the challenge of US population health improvement. With a prevalence rate of 10.5% and ranking as the 7th leading cause of death, diabetes is a priority condition for improving the nation’s physical and economic health [1]. Diabetes has ranked highest among chronic diseases in US health care and public health spending [2]. In 2017, 1 in 7 health care dollars was attributable to diabetes and 1 in 4 health care dollars went to the care of a person with diabetes [3]. The COVID-19 pandemic further revealed the excess vulnerabilities that diabetes, as a premorbid condition, confers on health status and utilization [4].

The human and economic costs of diabetes are not distributed equally; racial and socioeconomic disparities in diabetes result in marginalized populations carrying excess disease burden in incidence, prevalence, morbidity, mortality, and utilization [1, 5, 6]. Observed patterns of diabetes disparities have not improved despite advances in diabetes diagnostics, therapeutics, and prevention [7, 8]. To achieve diabetes population health improvement, attention has turned to the role of social determinants of health (SDOH) as a contributor to diabetes inequities. The American Diabetes Association (ADA) formed a SDOH scientific review committee to conduct a comprehensive review of SDOH frameworks, definitions, and evidence of impact in diabetes, with the goal of understanding and advancing opportunities for diabetes population improvement through addressing SDOH [9].

The aims of this paper are to summarize findings from the ADA SDOH scientific review for definitions and constructs, to highlight the evidence-based review key findings on the impact of SDOH on diabetes, and to expand upon the ADA’s scholarly review by illustrating SDOH impact in the context of diabetes using Black/African Americans^1^ as a population exemplar. Finally, US national committee recommendations for addressing SDOH, including a health care response and actions on systemic racism and socioeconomic discrimination as root causes, are discussed.

## SDOH and Diabetes

In 2008, the World Health Organization (WHO) Commission on the Social Determinants of Health released a report introducing concepts and frameworks for understanding and mitigating the effects of SDOH [10]. The WHO set the stage for several subsequent US national committees and organizations to explore SDOH in the context of related phenomenon of racial and socioeconomic health disparities [11, 12] and goals of achieving health equity [13]. Definitions of the key terms, including health disparities [11], health equity [13, 14], and SDOH [15], are presented in Table 1. The WHO’s definition of SDOH [15] remains the most widely used definition of the construct.

**Table 1:** Definitions

Term	Definition
Health disparities	A particular type of health difference that is closely linked with social, economic, and/or environmental disadvantage. Health disparities adversely affect groups of people who have systematically experienced greater obstacles to health based on their racial or ethnic group; religion; socioeconomic status; sex; age; mental health; cognitive, sensory, or physical disability; sexual orientation or gender identity; geographic location; or other characteristics historically linked to discrimination or exclusion
Health equity	Equity is the absence of avoidable, unfair, or remediable differences among groups of people, whether those groups are defined socially, economically, demographically, or geographically or by other means of stratification. “Health equity” or “equity in health” implies that ideally everyone should have a fair opportunity to attain their full health potential and that no one should be disadvantaged from achieving this potential Health equity is attainment of the highest level of health for all people. Achieving health equity requires valuing everyone equally with focused and ongoing societal efforts to address avoidable inequalities, historical and contemporary injustices, and the elimination of health and health care disparities
Social determinants of health^1^	Social determinants of health are the conditions in which people are born, grow, live, work and age, and also includes the health system. These circumstances are shaped by the distribution of money, power and resources at global, national and local levels, which are themselves influenced by policy choices. Social determinants of health are mostly responsible for health inequities – the unfair and avoidable differences in health status seen within and between countries

Reprinted and modified with permission from the following: Hill-Briggs F, Adler NE, Berkowitz SA, Chin MH, Gary-Webb TL, Navas-Acien A, Thornton PL, Haire-Joshu D. Social Determinants of Health and Diabetes: A Scientific Review. Diabetes Care 2021 Jan; 44(1): 258–279

^1^The World Health Organization’s 2021 modified definition of social determinants of health

The ADA SDOH scientific review examined SDOH frameworks and nomenclatures from the WHO [15, 16], Healthy People 2020 and 2030 [17], the County Health Rankings Model [18, 19], and Kaiser Family Foundation [20], identifying shared SDOH domains among the frameworks to comprise the SDOH for the literature review in diabetes. The five reviewed SDOH domains are socioeconomic status, housing and physical environment, food environment, health care, and social context [9]. These five SDOH and their component factors and descriptors are shown in Table 2. Highlights of the review findings are described. The reader is referred to the full ADA scientific review for detailed findings [9].

**Table 2:** Reviewed social determinants of health with their component factors and descriptors

Social determinant	Component factors	Description
Socioeconomic status	Education	Educational attainment, a measure of *quantity* of education received (years of education or highest degree earned by an individual, a household, or census tract in which a person resides). Educational achievement, in contrast, is a measure of *quality* of education (e.g., a person’s literacy skills)
	Income	Economic status, measured as a person’s own income, the income of the household, or the income level of the community (e.g., mean household income of the census track in which a person resides)
	Occupational status	Employment status or occupation/job category of a person
Neighborhood and physical environment	Housing	Housing instability, which comprises a range of situations including having trouble paying rent, experiencing evictions, frequent moves, living in one’s car, staying with relatives/friends, and homelessness
	Built environment	Characteristics of physical spaces, including infrastructure, buildings, open areas and green spaces, streets and walkability
	Toxic environmental exposures	Exposures that are either naturally occurring in the environment (e.g. arsenic) or produced by human activity (e.g. pollution and synthetic pesticides) that adversely affect health
Food environment	Food security	Within the food environment, having adequate quantity and quality of food at all times for all household members to have active, healthy lives
	Food access	Proximity to food sources and ability to reach food sources, measured, for example, as households with no car and living more than one mile from a grocery store
	Food availability	Number and distribution of food stores, including fast-food restaurants, full-service restaurants, grocery stores, convenience stores, and per capita sales in dollars from local farms made directly to consumers
Health care	Access	Ability to gain entrée to health care, measured as insured/uninsured status and availability of health care services and providers within one’s geographic location
	Affordability	Ability to bear the financial costs of health care services and therapeutics
	Quality	The extent to which care results in desired health outcomes and aligns with professional standards, measured as achievement of identified quality and performance measures
Social context	Social cohesion	The degree of connectedness and solidarity among groups in a society, and inclusivity versus marginalization of groups
	Social capital	Features of social structures that serve as resources for collective action (e.g., interpersonal trust, reciprocity norms, and mutual aid)
	Social support	Experiences in individuals’ formal and informal personal relationships as well as their perceptions of those relationships, in the areas of emotional support, tangible support, informational support, and companionship

Hill-Briggs F, Adler NE, Berkowitz SA, Chin MH, Gary-Webb TL, Navas-Acien A, Thornton PL, Haire-Joshu D. Social Determinants of Health and Diabetes: A Scientific Review. Diabetes Care 2021 Jan; 44(1): 258–279. PMID: 33139407

Socioeconomic status (SES) comprises education, income, and occupational status, which are intercorrelated but have unique associations with health. In the USA, type 2 diabetes is associated with lower SES. Diabetes prevalence in adults increases with lower educational attainment. In 2018, diabetes prevalence was 7.5% among adults with more than high school education, 9.7% among adults with a high school education, and 13.3% among adults with less than high school education [1]. Similarly, diabetes prevalence increases on a gradient with lower income, and the pattern of increasing diabetes prevalence with lower income gradients is observed within all racial/ethnic groups [21]. Having less than a high school education has been found to confer a diabetes mortality risk twice that of persons with a college degree, and persons living in poverty have a risk of diabetes mortality 2.4 times that of persons with an income ≥ 400% the federal poverty level, independent of age, sociodemographics, and BMI [22].

Within the neighborhood and physical environment SDOH, among adults with diabetes who receive care in community health centers, housing instability may be as high as 30%, and self-reported housing instability has been associated with higher frequency of diabetes-related emergency department visits and hospitalizations [23]. Higher exposure to green space and greater neighborhood walkability has consistently been shown to be associated with lower diabetes incidence and prevalence and better health outcomes [24]. Marginalized communities in the USA have higher exposure to toxic environments due to factors including closer proximity to sources of pollution; exposure to wells containing arsenic, other metallic alloys, and pesticides; and exposure to chemicals known to be endocrine disrupters (e.g., phthalates, mercury) through sources such as plastic food wrapping from fast foods, canned goods, and specific cosmetic products [25]. Food environment factors such as greater access to food, higher availability of full-service restaurants, higher density of grocery stores, lower exposure to fast-food outlets and convenience stores, and better both walkability and food availability are associated with lower type 2 diabetes incidence and prevalence and better glycemic control [26–28].

Studies of health care as a SDOH reveal that insurance status is the strongest predictor of whether or not an individual has access to diabetes screenings and care. Being uninsured or underinsured is associated with higher rates of undiagnosed diabetes, receipt of infrequent and lower quality of care, higher rates of emergency department visits, and higher A1C [9]. Geographic location, or place, determines access to endocrinologists and diabetes self-management education programs, with these diabetes services concentrated in higher SES communities [29, 30]. Documented racial disparities in diabetes care access, availability, and quality are associated with SES, neighborhood and place factors, and racial bias and discrimination [9, 31].

A relative paucity of research has examined social context factors of social capital and social cohesion and diabetes, but the few studies to date reveal that higher levels of social capital and cohesion, rather than marginalization or exclusion, are associated with lower diabetes incidence and better glycemic control [9, 28, 32]. There is evidence of associations of greater social support with better diabetes outcomes and quality of life and associations of lower social support with higher rates of diabetes complications and mortality [33–35].

The ADA review concludes that there is strong evidence linking the SDOH of SES, neighborhood and physical environment, food environment, and health care with diabetes incidence, prevalence, management, mortality, and disparities. Consequently, there is a need for effective approaches to intervening on these factors to improve diabetes population health overall and racial and socioeconomic inequities in diabetes specifically. Recommendations are enumerated to improve clarity in intervention directions, targets, and partnerships [9, 36].

## Illustrating the SDOH: Black/African Americans as a Population Exemplar in Diabetes

To illustrate the scope and impact of SDOH in the context of diabetes, and of racial disparities in diabetes and in SDOH factors, Black/African Americans are used as a population exemplar. Although race is a social construct, not a biological one [37, 38], in the context of SDOH, the social construct of classifying and assigning value to people based on the color of their skin has import and serves as a proxy for racial discrimination [20, 39].

### Racial Disparities in Diabetes Prevalence, Morbidity, and Mortality

Longstanding and pervasive patterns of disparities exist between Black/African Americans and White Americans across health conditions including, but not limited to, infant and maternal mortality, asthma, cancer, HIV/AIDS, hypertension, stroke, coronary heart disease, and diabetes [40]. Racial disparities in diabetes outcomes and in diabetes quality measures, specifically, persist despite advances in diabetes therapeutics [7, 41]. A sample of diabetes disparities is presented in Table 3 to illustrate such patterns [1, 42–48]. Black/African American adults are 73% more likely to have diabetes (diagnosed and undiagnosed) than White adults [42] and more than twice as likely to die from diabetes-related causes [1]. NHANES data from 1999 to 2018 indicate that Black/African American adults with diagnosed diabetes are significantly less likely than their White counterparts to achieve diabetes clinical and quality targets [41]. Black/African American adults with diabetes are 1.5 to 2.5 times more likely to have complications of diabetic retinopathy, lower limb amputation, major CVD, stroke, and ESRD than are White adults [43–45, 48] and almost 3 times more likely to be hospitalized for uncontrolled diabetes and short-term complications of diabetes [45]. As a SDOH, the context of health care and care provision have contributed to racial/ethnic minorities and lower SES groups experiencing lower health care quality, patient experience, and outcomes, as documented in the Institute of Medicine 2003 report, *Unequal Treatment* [31]. In addition to access and affordability issues, inequities discussed in the report, including implicit racial bias influencing provider attitudes, communication and relationship-building, clinical decision-making, and prescribing behaviors, continue to be observed as mechanisms by which these inequities continue [49–51].

**Table 3:** Examples of racial disparities in diabetes outcomes

Diabetes outcome	Black/African Americans	White Americans
Incidence [1]		
Age-adjusted incidence diagnosed diabetes (per 1,000 adults)	8.2	5.0
Prevalence [42]		
Diagnosed diabetes	13.6%	9.1%
Undiagnosed diabetes	4.7%	4.3%
Total with diabetes	18.3%	13.3%
Prevalence of clinical and quality target achievement [41]		
A1C < 8.0%	68.7%	76.7%
Individualized A1C goal	60.4%	68.3%
Blood pressure < 140/90 mmHg	61.3%	72.7%
Blood pressure < 130/80 mmHg	38.7%	48.5%
LDL cholesterol < 100 mg/dL	46.9%	56.6%
Individualized A1C goal + Blood pressure < 130/80 mmHg + LDL cholesterol < 100 mg/dL	12.5%	20.6%
Mortality [47]		
Age-adjusted diabetes death rate (per 100,000)	39.3	18.9
Diabetes-related complications		
Age-adjusted incidence end-stage renal disease (per million population) [45]	366.2	138.4
Chronic kidney disease [44]	26.0%	24.0%
Diabetic retinopathy [48]	38.8%	26.4%
Coronary heart disease [43]	16.3%	23.1%
Hospitalization for stroke (per 1,000 diabetic adults) [43]	11.5	7.4
Hospitalization for major CVD (per 1,000 diabetic adults) [43]	66.8	44.3
Hospital admission for lower extremity amputations (per 1,000 diabetic adults) [45]	60.9	26.8
Hospital admission for short-term complication of diabetes (per 100,000 adults) [45]	141.5	55.4
Hospital admission for uncontrolled diabetes without complications (per 100,000 adults) [45]	114.1	36.4

### Racial Inequities in Social Determinant Factors

At the population level, conditions in which Black/African Americans are born, grow, live, work, and age differ from those of White Americans, and inequality in these SDOH conditions has been shaped by historical inequities. Table 4 displays several key SDOH indicators by way of example [52–65]. Ogunwole and Golden [66] depict an explanatory model, based on Nicholas, Kalantar-Zadeh, and Norris [67], in which racism is the root cause of socioeconomic deprivation, manifested in the seminal conditions of residential segregation, discrimination, and lack of insurance/underinsurance, which each in turn have caused a cascade of health, social, and environmental injustices and vulnerabilities underlying observed health disparities in the USA.

**Table 4:** Examples of racial inequities in social determinants of health

Social determinant of health	Black/African Americans	White Americans
Educational attainment (quantity)		
High school or more [52]	89.4%	91.3%
Bachelor’s degree [52]	27.8%	37.5%
Advanced degree [65]	9.9%	14.0%
Educational achievement (quality) [53]		
Proficient literacy	23%	58%
Basic or below basic literacy	75%	42%
Income and wealth		
Median household income [57]	$45,870	$71,231
Population below poverty [57]	19.5%	8.2%
Population 125% below the poverty line [57]	25.6%	11.0%
Median wealth [56]	$24,100	$188,200
Employment and occupation		
Unemployment rate [54]	11.4%	7.3%
Occupation [55]		
Management, professional, and related occupations	9.7%	78.7%
Janitors, building cleaners	17.0%	74.7%
Baggage porters, bellhops	24.6%	61.1%
Means of transportation to work: Public transportation [63]	11.1%	3.1%
Workers without a vehicle at home [63]	9.5%	2.8%
Neighborhood and housing		
Among residents in high poverty neighborhoods/census tracts [58]	20%	4%
Among residents in extreme poverty neighborhoods/census tracts [62]	25.2%	7.5%
Home ownership rate [59]	45.3%	71.3%
Mortgage applications denied rate [60]	18.1%	6.9%
Among homeless persons [78]	39.4%	48.3%
Among homeless families with children [78]	53.1%	35.0%
Food environment [61]		
Food insecurity	21.7%	7.1%
Very low food insecurity	8.0%	3.0%
Health care [64]		
Working-age adults without health insurance coverage	14.2%	9.0%
Working-age adults without health insurance coverage, expansion state^1^	10.2%	6.9%
Working-age adults without health insurance coverage, non-expansion state	18.9%	13.0%

^1^Expansion states are those that expanded Medicaid by January 1, 2019. As of that date, there were 17 states that had not yet expanded Medicaid

Examining Black/African Americans, education trend data from 1940 to 2020 show that, although attainment of high school education is reaching parity [52, 65], stark racial disparities remain in educational quality, as measured by literacy proficiency. In 2012–2014, 23% of Black/African Americans and 58% of White Americans had proficient literacy [53]. Historically, US state anti-literacy laws, which made it illegal for Black/African American persons, whether enslaved or freed, to be taught to read or write or to assemble for the purposes of teaching or education, persisted in some states until the 1930s [68]. Not until Brown vs. the Board of Education [69] was the policy of racial segregation in US education declared to deprive racial and ethnic minority children the educational opportunities afforded to White children in public schools, with actual desegregation of public and private schools lagging by decades [70]. In the present era, patterns of neighborhood and school segregation persist, and school districts that are predominantly White receive more funding and are better resourced than are districts comprising predominantly racial/ethnic minority children [71].

In the setting of educational inequities, racial differences are also observed in employment and occupational opportunities, and consequently, in income and wealth [40, 54]. Moreover, in 2020, the unemployment rate was 13.2% for Black/African Americans and 7.9% for White Americans [54]. Since emancipation from slavery and integration into a paid US workforce, Black/African Americans have been overrepresented in service-related occupations, such as janitors, baggage porters, and food services [55]. Racial gaps in wages adversely impact earnings of Black/African American workers, and Black women are at the intersection of race and gender wage gaps [72, 73]. While White women are paid 82% of what White men are paid, Black/African American women are paid only 63% [74]. With hundreds of years of disproportionate accumulation of wealth due to the institution of slavery, today US income and wealth data, by race, remain markedly disparate [56]. In 2021, Black/African Americans had a median household income of $45,870 USD compared with $71,231 for White Americans [57]. One in four Black/African American individuals lives below poverty, compared with one in twelve White American individuals, and among individuals 125% below poverty line, 67.9% are Black/African American [57].

The neighborhood and housing environment of Black/African Americans as a population, compared with the White population, reflects persisting patterns of inequity resulting from US policies and practices of residential segregation by race, redlining, and zoning and access restrictions that in turn determine the food environment and exposures to toxicity for Black/African Americans [66, 75–77]. Among persons residing in high poverty neighborhoods/census tracts in the USA (Table 4), 1 in 5 is African American, and among those residing in extreme poverty neighborhoods, 1 in 4 is Black/African American [58, 62]. Home ownership remains low among Black/African American adults [59, 60], due to inequities in income, occupation, and wealth; residential segregation practices; and mortgage denial rates. In 2020, although Black/African Americans comprised 12% of the US population, they comprised 39% of the homeless population, as compared with White Americans, who comprised 74% of the US population and 48% of the homeless population [78]. Inequity in access to food is also reflective of the patterns in income, poverty, and neighborhood environment, with the Black/African American population experiencing food insecurity at three times the rate of the White population [61].

## Intervening on the SDOH

### Recommendations for a Health Care Response to SDOH

US national committees and agencies have published recommendations for the health sector and health care organizations to address SDOH. These recommendations include National Academies of Sciences, Engineering, and Medicine (NASEM) reports on educating health care professionals to take action on SDOH [79], integrating social needs care into health care delivery [80], and addressing SDOH as a component of high-quality, patient-centered primary care [81], and United States Preventive Services Task Force (USPSTF) reports [12, 38], among others. Several recommendations and resources address assessment and measurement of SDOH at both the individual patient level and community and population levels, as well as needs for data infrastructure for SDOH monitoring and evaluation [80, 82–84]. It is important to note that there is not consensus regarding items that constitute a SDOH assessment for planning SDOH interventions or responding to patients’ social needs individually or collectively. Current assessment tools recommended as SDOH measures tend to combine SDOH, health behaviors, and behavioral risk factors (e.g., physical activity, tobacco use), as well as clinical disease (e.g., substance abuse, depression). Consequently, careful consideration is needed to align appropriate workforces to conduct assessment and appropriate intervention pathways for social needs, which may be different from those needed to improve health behaviors and clinical disease. In addition to US committee recommendations, there are specific resources designed to aid health care professionals and organizations, at the clinical level, identify social needs tools and services for referrals, as highlighted in the ADA SDOH review [9].

Within health care and public health sectors, a body of health disparities research has yielded interventions and care models that demonstrate effectiveness in improving intermediate outcomes in individuals directly served by the intervention [5, 36, 85]. These interventions often augment traditional health care delivery through ancillary workforces or community settings of care, or they modify or adapt interventions and resources for accessibility, cultural relevance, suitability, and acceptability. Reviews of the health disparities intervention literature in diabetes specifically include various community health worker interventions for home- and community-based risk factors and symptom monitoring, health education, and instrumental and social support; navigation interventions for clinical care and for neighborhood-based health and social services; and interventions to address food deserts and food insecurity through partnerships with food banks or grocery delivery services [5, 85, 86]. Methods to compensate for lower literacy and health literacy skills resulting from poor educational quality or attainment have also been used in diabetes care and are available to facilitate patients’ understanding and use of health information and suitability of educational and behavioral interventions [87–89]. These sets of intervention approaches are key to meeting immediate needs of the individuals who receive the intervention. However, the limitation in the scope of such interventions is that they do not provide long-term problem resolution of the underlying and likely persistent social determinant conditions [36, 66].

### Addressing Root Causes of SDOH

Effectively mitigating the SDOH is challenging because SDOH are systemic, population-based, cyclical, and intergenerational in nature. Root cause recommendations address systemic, structural, and historic causes of SDOH. The WHO recommendations on ameliorating SDOH focus on actions to change social, economic, and political systems and dynamics as root causes of SDOH. Three specific recommendations are presented in the 2008 Commission report on SDOH. First, improve daily living conditions by emphasizing early childhood education and development, better working conditions, and social protection for all. Second, tackle the inequitable distribution of power, money, and resources by creating a strong, competent, and adequately financed public sector, and committing to reinvestment in collective benefit efforts and to having an accountable private sector. Third, measure and understand the problem and assess the impact of actions taken, by acknowledging the health equity problem, using surveillance systems for routine monitoring of SDOH and inequities, and evaluating the multi-level effects of policies and interventions [10, 16].

The USPSTF affirms that SDOH have a strong influence on personal health and that having evidence-based preventive recommendations that address SDOH would be of strong benefit [90]. However, several challenges impede this goal, including non-consensus regarding proposed SDOH, lack of available evidence on preventive interventions for SDOH, and need for clarity regarding responsible organizations and agencies [90]. To inform further inclusion of SDOH considerations within its methodology, the USPSTF examined how social risk has been considered in its 85 active recommendations since 2019, with a focus on Centers for Medicare & Medicaid Services Accountable Health Communities social risk domains of housing and food insecurity, transportation difficulties, utility assistance needs, interpersonal safety, education, and financial strain [12]. The review revealed that 57 of the 85 recommendations contained comments on social risk, either appearing within risk assessments, as considerations for clinicians in determining worsening disease risk or for implementation of preventive services, or as research gaps [12].

The CDC has compiled resources designed to support action on SDOH, including tools that identify policy and multisector partnership opportunities to address priority SDOH [91], and best practices, guides, and toolkits to enable moving from planning to taking action on SDOH [92]. The American Public Health Association (APHA) and the CDC endorse Health in All Policies (HiAP) as a means to address SDOH [93, 94]. Several key features characterize the HiAP approach. Among those presented in the APHA’s HiAP 2013 report are:

“(1) Health in All Policies is a collaborative approach to improving the health of all people by incorporating health considerations into decision-making across sectors and policy areas. (2) Health is influenced by the social, physical, and economic environments, collectively referred to as the “social determinants of health.” (3) Health in All Policies, at its core, is an approach to addressing the social determinants of health that are the key drivers of health outcomes and health inequities.” [94]

HiAP represents a comprehensive response to the presence of SDOH in all sectors and the need, therefore, for all sector considerations, action plans, and policymaking to be mindful of SDOH. Consistent with HiAP is the WHO framework for inclusion of health equity as a goal in all health and social policies in order to tackle SDOH inequities [16].

Health care policies are an example of interventions that can change the social determinant conditions. Insured status is a key racial inequity and opportunity for intervention [64]. With the expansion of Medicaid under the Affordable Care Act (ACA), for example, the uninsured rate for black adults aged 19–64 years dropped 9 percentage points in the first 2 years, reducing the Black-White disparity in lack of health insurance from 10 to 6 percentage points [95]. Among states that have failed to adopt the ACA Medicaid expansion (e.g., non-expansion states), disparities in health care access, utilization, and outcomes persist [96]. In a comparison of hypertension and diabetes outcomes among patients in expansion versus non-expansion states, patients in expansion Federally Qualified Health Centers (FQHC) experienced a 3.38 percentage point improvement in hypertension control and 3.88 percentage point improvement in diabetes control among Black patients [97].

### Racism as a Root Cause of SDOH

Racism is increasingly acknowledged in the USA as a public health issue and as a root cause of health inequities [38, 98], with socioeconomic factors as downstream consequences of racism for marginalized populations [66, 67]. Racism is defined by the CDC as “a system–-consisting of structures, policies, practices, and norms—that assigns value and determines opportunity based on the way people look or the color of their skin” [98]. Recently, the American Psychological Association (APA) issued a statement regarding the psychology discipline and organization’s participation and complicity in racial discrimination historically [99], along with statements regarding APA’s commitment to health equity [100] and to dismantling systemic racism against people of color in the USA [101].

APHA has declared racism a public health crisis and has released several statements related to the impact of racism on health [102], as well as an interactive tool and analysis of resolutions declaring racism as a public health crisis across the country [103, 104]. As of August 2021, 209 declarations of racism as a public health issue were passed in 37 states [103]. The first jurisdictions in the country to pass resolutions declaring racism a public health crisis were Milwaukee, WI, in 2018 and Pittsburgh, PA, in 2019; soon after many communities did so, largely in response to the 2020 police killing of George Floyd. The New York City Health Department is one of the most recent jurisdictions to declare [105]. The content of the declarations and resolutions include themes such as systemic racism, SDOH, specific health outcomes, and COVID-19’s disproportionate impact on Black, Indigenous, and Latinx communities [106]. Strategic actions associated with the declarations widely vary. For example, some of the declarations have suggested creating an office, group, or taskforce to collect data on racial inequities and ensure accountability on equity goals. Others have focused data and accountability, placing emphasis on improving the collection, analysis, and reporting of data on SDOH and/or disaggregated racial and ethnic data. There are also suggested strategic actions in the areas of community engagement, policies and programs, organizational capacity/training, and funding. Of the jurisdictions who have declared racism as a public health issue, few have committed to specific strategic actions or funding [106].

The USPSTF aims to address health inequities caused by systemic racism through a process of transforming the methods used by the task force in developing recommendations [38]. By transforming the recommendation-making process, the USPSTF intends to mitigate the influence of systemic racism in the recommendations it puts forth. Doubeni, Simon, and Krist [38, 107] describe six action steps to be implemented: (1) consider race as a social, not a biological construct, (2) promote racial and ethnic diversity in USPSTF membership and leadership and foster a culture of inclusivity, (3) commission a report to understand how systemic racism undermines the benefits of evidence-based clinical preventive services, (4) iteratively update methods to overcome health inequities experienced by populations affected by systemic racism, (5) communicate gaps created by systemic racism in all dissemination efforts, (6) collaborate with its partners and experts to reduce the influence of systemic racism on health. Moreover, the USPSTF highlights the key roles of partners across sectors in disseminating and implementing USPSTF recommendations and reducing health inequities through mitigating influences of racism [38]. Next, the USPSTF published an audit of all frameworks, policy, and position statements addressing racism, along with a systematic review of interventions that reduce health inequities or prevent racism [38, 68]. Finally, the USPSTF published an official policy on all of its changes aimed at reducing health inequities and racism, including prioritizing topics based on their impact on these two public health crises [38, 68].

The emerging naming of racism as a root cause contributor to SDOH and to longstanding patterns of health disparities in the USA enables movement toward non-traditional partnerships and initiatives to tackle the challenge of intervening to improve the population’s health.

## Conclusions

There is strong evidence that SDOH, including socioeconomic status, neighborhood and physical environment, food environment, health care, and social context inclusive of racism, are associated with diabetes risk and outcomes and with observed diabetes disparities. Because the SDOH are systemic, population-based, cyclical, and intergenerational, intervening to mitigate the SDOH requires extension beyond health care solutions to multi-sector and multi-policy approaches to achieve future population health improvement. Committing to actions that reduce health inequities, mitigate systemic racism, and improve the health of those at risk or with diabetes is needed now.

## Footnotes

1 In this paper, when referring to racial groups, the terms “Black” and “White” are capitalized in accordance with APA standards for bias-free language when referring to racial and ethnic minority groups. See https://apastyle.apa.org/style-grammar-guidelines/bias-free-language/racial-ethnic-minorities. “*Racial and ethnic groups are designated by proper nouns and are capitalized. Therefore, use ‘Black’ and ‘White’ instead of ‘black’ and ‘white’ (do not use colors to refer to other human groups; doing so is considered pejorative). Likewise, capitalize terms such as ‘Native American,’ ‘Hispanic.’*”.
